# Ameliorative Potential of Hydroethanolic Leaf Extract of *Parquetina nigrescens* on d-Galactose-Induced Testicular Injury

**DOI:** 10.3390/molecules26113424

**Published:** 2021-06-05

**Authors:** Lydia Ajayi, Ademola Ayeleso, Temitope Oyedepo, Emmanuel Mukwevho

**Affiliations:** 1 Department of Biochemistry, Faculty of Science, Adeleke University, P.M.B. 250, Ede 232001, Nigeria; ajayity82@gmail.com (L.A.); ademola.ayeleso@adelekeuniversity.edu.ng (A.A.); topeoyedepo@adelekeuniversity.edu.ng (T.O.); 2 Department of Biochemistry, Faculty of Natural and Agricultural Science, Mafikeng Campus, North West University, Mmabatho 2735, South Africa

**Keywords:** *Parquetina nigrescens*, d-galactose, toxicity, oxidative stress, inflammation, apoptosis

## Abstract

Background: There is an increasing need for botanicals to be used as an alternative and complementary medicine in the management of male infertility. Male infertility has been a major health/social challenge to people all over the world. This study, therefore, investigated the ameliorative potential of hydroethanolic leaf extract of *Parquetina nigrescens* (HELEPN) against d-galactose-induced testicular injury. Methods: Thirty male Wistar rats were randomly allotted into six groups (n = 5). Group I (Normal control), Group II (300 mg/kg b.w. d-galactose), Group III and IV (250 and 500 mg/kg b.w. HELEPN, respectively), Group V and VI (both received 300 mg/kg b.w. of d-galactose with 250 and 500 mg/kg b.w of HELEPN, respectively). d-galactose administration started two weeks prior to HELEPN treatment which lasted for six weeks. All assays were carried out using established protocols. Results: Administration of HELEPN at 250mg/kg and 500mg/kg concomitantly with d-galactose improved paired and relative testicular weights, levels of gonadotropins (LH and FSH) and testosterone, and poor sperm quality. HELEPN treatment reduced the levels of oxidative stress biomarkers (MDA, 8-OHDG, and AGEs) and inflammatory response (TNF-alpha and NO) to normal, as well as restoring the reduced activities of antioxidant enzymes (glutathione peroxidase, superoxide dismutase, and catalase). In addition, HELEPN treatment mitigated testicular DNA fragmentation and down-regulated caspase 3-activities. HELEPN at 500 mg/kg was observed to have the greatest ameliorative effect. Conclusion: HELEPN protects against d-galactose-induced testicular injury through antioxidative, anti-inflammatory, and antiapoptotic mechanisms.

## 1. Introduction

*Parquetina nigrescens* is a shrub that belongs to the family *Asclepiadaceae* [1]. It is abundant in West Africa and useful in herbal medicine for treating diseases. The plant is known as African parquetina in the English language, *Kwankwani* in hausa, *mgbidingbe* in Igbo, and *Ewe ogbo* in Yoruba [2,3]. It is a perennial plant that has a spiraling stem, a ligneous base, and is about 10–15 cm long abd 6–8 cm broad with a long smooth stem that bears the leaves [4]. Over the years in East Africa, the leaf decoction of the plant has been used as an aphrodisiac in folk medicine [5,6]. Improvement of sexual activities as a result of administration of aqueous extract of *P. nigrescens* on paroxetine-induced sexual dysfunction has been shown through changes in reproductive hormones, nitric oxide, and activity of phosphodiesterase V in male rats [3].

*P. nigrescens* contains various phytochemicals such as flavonoids, phenols, alkaloids, saponins, tannins, phlobatannins, anthraquinones, triterpenes, cardenolides, reducing sugar, cardiac glycosides, steroid, and terpenoid [3,7]. Generally, phytochemicals from plants are known to possess therapeutic properties for managing various diseases [8]. *P. nigrescens* possess analgesic, anti-inflammatory, and antipyretic potentials [4], as well as anti-ulcerative, anti-helminthic, pro-hematopoietic, antidiabetic, and antioxidant properties [1,9].

The normal functioning of the reproductive system is dependent on proper sperm cell production. It is a cascade of events that include numerous factors such as the availability of pro-inflammatory cytokines, tumor necrosis factor-alpha, and Interleukin -1-alpha in the male reproductive tract [10]. However, a higher concentration of these cytokines, as seen in conditions of inflammation, could be detrimental to the process of sperm production. Moreover, there is a clear relationship between inflammation, apoptosis, and oxidative stress that have been associated with impaired sperm function. Epidemiological studies on male infertility have shown that a good number of infertile men suffer from acute or chronic inflammation [10]. Testicular DNA fragmentation is a vital etiological factor in male infertility. It is usually as a result of testicular oxidative DNA damage and apoptosis [11]. Testicular oxidative damage has been implicated in male infertility [12]. The peroxidative damage triggers apoptosis [13] with consequent impairment of testicular steroidogenesis as well as spermatogenesis [14]. Caspase 3 plays a vital role in the regulation of apoptosis in the seminiferous epithelium [15,16]. It has been identified in the development of impaired spermatogenesis and sperm quality and increased testicular/sperm DNA fragmentation [15]. Once activated, it stimulates the activity of endonuclease caspase-activated DNAse I (CAD) by cleaving CAD inhibitor (ICAD) [16], causing degradation of chromosomal DNA inside the nucleus, which leads to chromatin condensation and ultimately fragmented DNA and cell death [16].

Excess supply of d-galactose may result in ROS generation that causes mitochondrial dysfunction, oxidative stress, inflammation, and apoptosis in cells [17]. Elevated levels of d-galactose may be oxidized by galactose oxidase to produce hydrogen peroxide, which is a pro-oxidant and causes a decrease in enzymatic antioxidant, superoxide dismutase [18]. Furthermore, d-galactose can activate the onset of non-enzymatic glycation reactions to yield advanced glycation end products (AGEs) [19], which consequently react with their receptors (RAGE) to produce reactive oxygen species (ROS) through NADPH oxidase activation [20]. Galactose reductase also reduces d-galactose to form galactitol, which leads to osmotic stress [19]. Although the aphrodisiac effect of *P. nigrescens* has been reported [3], this study investigated the protective effect and the associated possible mechanism of action of hydroethanolic leaf extract of *P. nigrescens* against d-galactose–induced testicular damage.

## 2. Results

### 2.1. Chemical Constituents of HELEPN

The quantitative phytochemical analysis of the plant extract revealed the presence of alkaloids, saponins, tannins, cardiac glycosides, and anthraquinone, as shown in Table 1. Alkaloids were seen to have the highest quantity in the extract.

### 2.2. Effect of Graded Doses of HELEPN on Body Weight Gain, Paired Testicular Weight, and Relative Testicular Weight

Rats in all groups showed a significant reduction in body weight gain at *p* < 0.05. However, only d-galactose-treated rats had significantly lower paired testicular weight and relative testicular weight. Rats treated with 250 mg/kg and 500 mg/kg of HELEPN only and 250 mg/kg and 500 mg/kg HELEPN in combination with 300 mg/kg d-galactose had comparable paired testicular weight and relative testicular weight with the control group at *p* < 0.05 (Table 2).

### 2.3. Effect of Graded Doses of HELEPN on Sperm Analysis and Morphology

**Figure 1:**
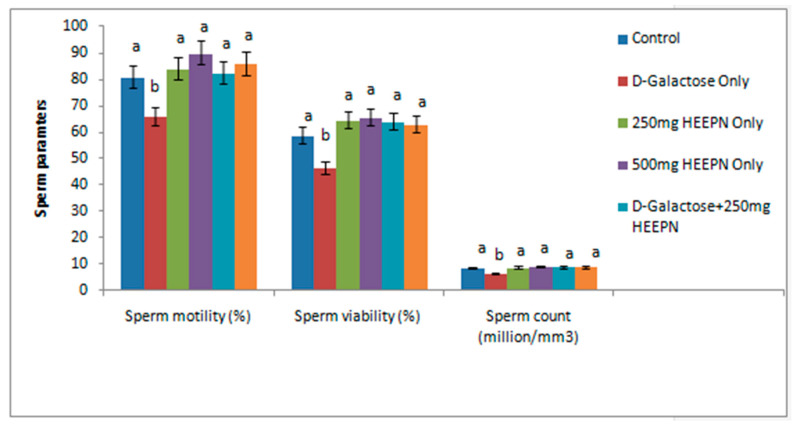
Effect of graded doses of hydroethanolic extract of *P. nigrescenson* sperm parameters. Values are means of five replicates ± SEM. Bars carrying different superscripts (a, b) are significantly different at *p* < 0.05.

d-galactose-treated rats had significantly lower sperm motility, viability, and sperm count (Figure 1) in comparison with the normal control group and other groups. However, these sperm parameters were restored in all animals that received HELEPN at 250 mg/kg and 500 mg/kg at *p* < 0.05. The changes observed in these parameters were not significantly different between the HELEPN-treated groups (when used singly or in combination with d-galactose) at 250 mg/kg and 500 mg/kg and the normal control group at *p* < 0.05. Similarly, sperm morphology was comparable in all groups compared with the control group except in the rats treated with d-galactose only, which had a higher percentage of abnormal sperm morphology compared with the normal control group (Table 3).

### 2.4. Effect of Graded Doses of HELEPN on Reproductive Hormones

**Figure 2:**
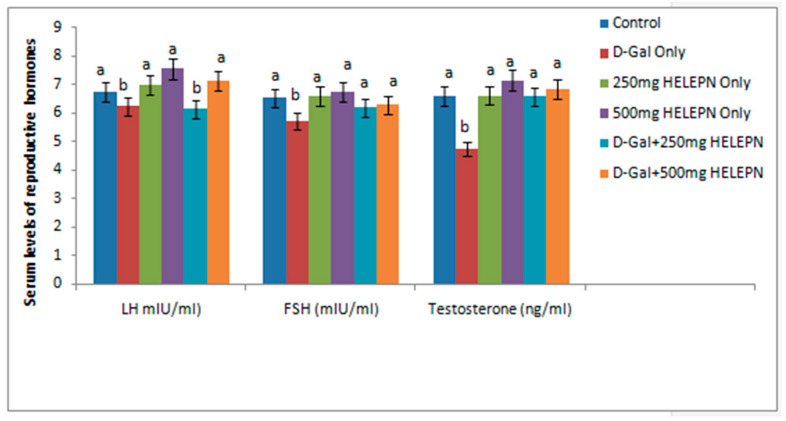
Effect of graded doses of HELEPNon reproductive hormones.Values are means of five replicates ± SEM. Bars carrying different superscripts (a, b) are significantly different at *p* < 0.05.

Serum FSH and testosterone were comparable in all with the controls, except in rats treated with d-galactose only, which hadlower concentrations of these reproductive hormones at *p* < 0.05. A significant reduction was observed in the levels of LH of the group given 250 mg/kg of HELEPN + d-galactose and the control group, but there was no significant difference between levels of LH of the control group and the group that was administered 500 mg/kg of HELEPN + d-galactose (Figure 2).

### 2.5. Effect of Graded Doses of HELEPN on Oxidative Stress Biomarkers

**Figure 3:**
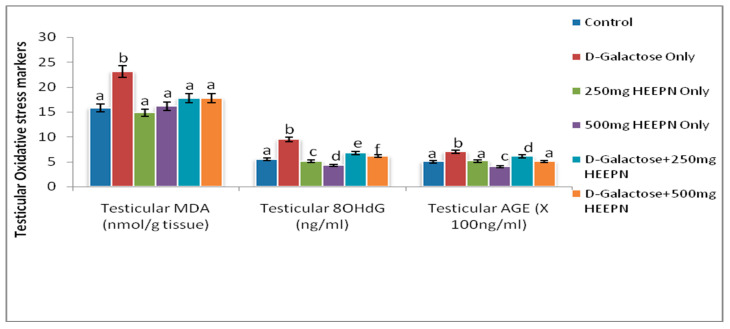
Effect of graded doses of HELEPN on oxidative stress biomarkers.Values are means of five replicates ± SEM. Bars carrying different superscripts (a–f) are significantly different at *p* < 0.05.

The testicular level of MDA was significantly higher in d-galactose-treated rats when compared with all other groups at *p* < 0.05 (Figure 3). Results from this study revealed that d-galactose caused a significantly increased level of oxidative DNA marker, 8-OHdG, when compared to all other groups at *p* < 0.05. HELEPN at 250 mg/kg and 500 mg/kg led to a significant reduction at *p* < 0.05 in 8-OHdG when compared with the control. The observed reduction in 8-OHdG in HELEPN-treated rats was dose-dependent. Similarly, HELEPN, when administered in combination with d-galactose at 250 mg/kg and 500 mg/kg, caused a marked reduction in 8-OHdG when compared with d-galactose only treated rats at *p* < 0.05 (Figure 3). Testicular AGEs was significantly increased in the group given d-galactose when compared with the normal control group and all HELEPN treated groups. The reduction of AGE level in the groups treated with d-galactose and HELEPN at 250 mg/kg and 500 mg/kg at *p* < 0.05 was found to be dose-dependent (Figure 3).

### 2.6. Effect of Graded Doses of HELEPN on Antioxidant Indices

**Figure 4:**
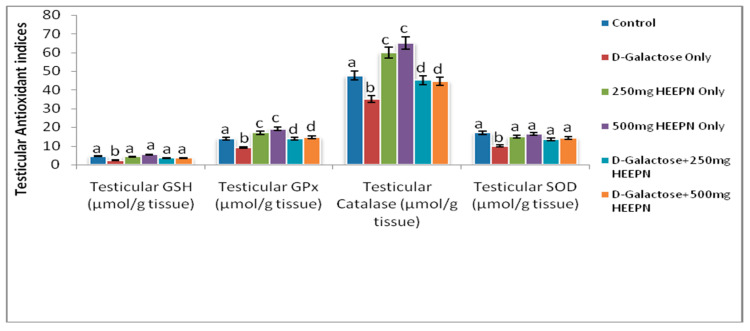
Effect of graded doses of HELEPN on antioxidant indices.Values are means of five replicates ± SEM. Bars carrying different superscripts (a–d) are significantly different at *p* < 0.05.

The testicular level of SOD was significantly reduced in d-galactose-treated rats when compared with all other groups at *p* < 0.05. Similarly, the testicular level of GSH was significantly reduced in d-galactose-treated rats when compared with all other groups at *p* < 0.05. Testicular activities of GPx and catalase were significantly lower in d-galactose-treated rats but higher in HELEPN-treated rats at 250 mg/kg and 500 mg/kg in comparison with the normal control group. Although the testicular activities of GPx and catalase were significantly higher in rats treated with the combination of d-galactose and HELEPN at 250 mg/kg and 500 mg/kg when compared with those treated with d-galactose only, their activities were close to that of the normal control animals at *p* < 0.05 (Figure 4).

### 2.7. Effect of Graded Doses of HELEPN on Inflammatory Biomarkers

**Figure 5:**
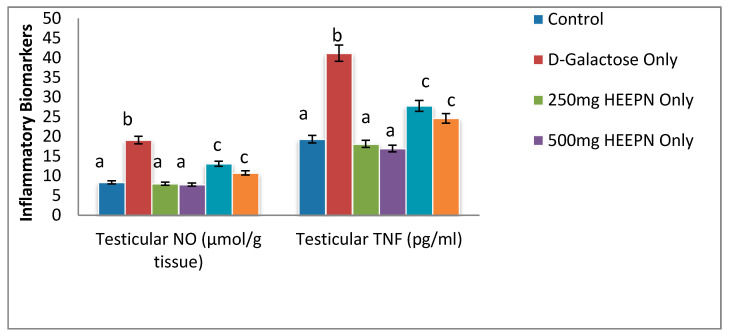
Effect of graded doses of HELEPN on inflammatory biomarkers. Values are means of five replicates ± SEM. Bars carrying different superscripts (a–c) are significantly different at *p* < 0.05.

Results from this study revealed that d-galactose led to increased testicular NO and TNF-α when compared to all other groups at *p* < 0.05 (Figure 5). Although HELEPN-treated rats at both 250 and 500 mg/kg had lower levels of testicular NO and TNF-α in comparison with the control, these alterations were not statistically significant at *p* < 0.05. When given in combination with d-galactose, HELEPN at 250 mg/kg and 500 mg/kg caused a significant decrease in testicular NO and TNF-α when compared with d-galactose-treated rats in a dose-dependent manner.

### 2.8. Effect of Graded Doses of HELEPN on Apoptotic Biomarkers

Testicular caspase 3 activity was similar in all groups except in d-galactose treated rats that had higher activity of caspase 3 at *p* < 0.05 (Table 4). HELEPN, when administered alone at 250 mg/kg and 500 mg/kg, led to reduced testicular DNA fragmentation. The HELEPN-induced reduction in testicular DNA fragmentation was not statistically significant at *p* < 0.05. d-galactose led to significantly higher testicular DNA fragmentation when compared with the control at *p* < 0.05. HELEPN, when administered in combination with d-galactose at 250 mg/kg and 500 mg/kg, caused a significant reduction in testicular DNA fragmentation to near normal (Table 4).

### 2.9. Histological Evaluation of the Effect of Graded Doses of HELEPN on the Testis

**Figure 6:**
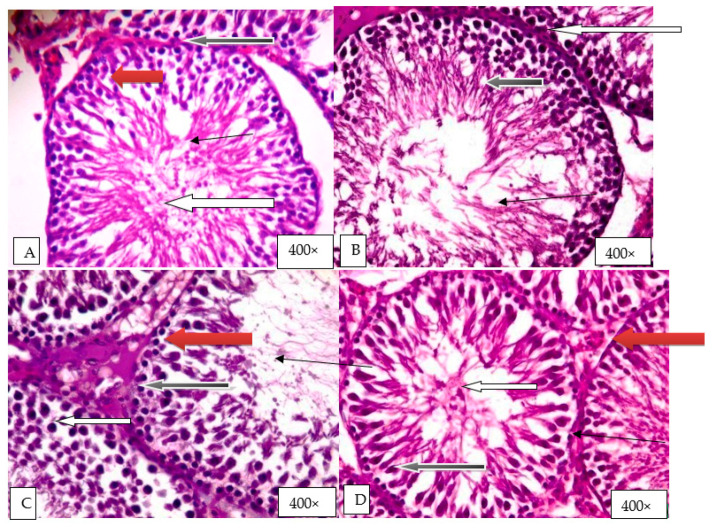

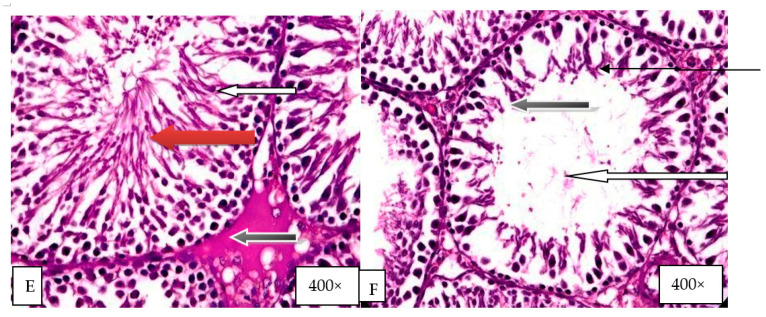
Histological evaluation of the effect of graded doses of HELEPN on the testis. Group (**A**) (control), Group (**B**) (d-galactose), Group (**C**) (250 mg/kg HELEPN), Group (**D**) (500 mg/kg HELEPN), Group (**E**) (d-galactose and 250 mg/kg HELEPN), Group (**F**) (d-galactose and 500 mg/kg HELEPN).

Group A (control) revealed normal testicular architecture with normal seminiferous tubules and normal maturation stages, showing the presence of sperm cells within their lumen (white arrow) (Figure 6). The seminiferous tubules were enveloped by normal connective tissue propria, lined inward by normal spermatogonia (blue arrow) and normal Sertoli cells (red arrow). Normal Leydig cells were also seen in the interstitial spaces (slender arrow). Group B (d-galactose) showed few normal seminiferous tubules that had normal germ cells and normal maturation stages, showing the presence of sperm cells inside their lumen (white arrow). Few seminiferous tubules showed atrophy, vacuolation, and maturation arrest (black arrow). Normal Leydig cells were also seen in the interstitial spaces (slender arrow) (Figure 6). Group C (250 mg/kg HELEPN) showed normal testicular architecture with normal seminiferous tubules and normal maturation stages with the presence of spermatozoa within their lumen (white arrow).The interstitial spaces showed normal Leydig cells (slender arrow) (Figure 6). Group D (500 mg/kg HELEPN) showed normal testicular architecture with normal seminiferous tubules and normal maturation stages with the presence of spermatozoa within their lumen (white arrow). The interstitial spaces showed normal Leydig cells (slender arrow) (Figure 6). Group E (d-galactose and 250 mg/kg HELEPN) showed some normal seminiferous tubules containing normal germ cells and normal maturation stages with the presence of spermatozoa within their lumen (white arrow) (Figure 6). Some seminiferous tubules showed obvious maturation arrest (black arrow).The interstitial spaces showed normal Leydig cells (slender arrow). Group F (d-galactose and 500 mg/kg HELEPN) showed several normal seminiferous tubules containing normal germ cells and normal maturation stages with the presence of spermatozoa within their lumen (white arrow). Very few seminiferous tubules showed maturation arrest at the secondary developmental stage (black arrow).The interstitial spaces showed normal Leydig cells (slender arrow) (Figure 6).

## 3. Discussion

This study elucidated the potentials of hydroethanolic leaf extract of *Parquetina nigrescens* (HELEPN) to abrogate testicular toxicity caused by d-galactose in male rats. The extract of *Parquetina nigrescens* has been established not to be toxic at 1600 mg/kg for six weeks in rats [21] and based on this, it is unlikely to be unsafe for human consumption. Induction of inflammation and oxidative stress by d-galactose has been well established [22]. In addition, the effect of aqueous extract of *P. nigrescens* on sexual competence has been documented [3], however, this study seems to be the first to investigate the effects of hydroethanolic extract of *P. nigrescens* on testicular injury.

Administration of d-galactose resulted in an increased level of tumor necrosis factor-ᾳ, which is majorly produced by the testicular macrophages in the gonads. During tissue damage, the movement of leucocytes into tissues is determined mainly by TNF-alpha, and therefore, made TNF-ᾳ contributes largely to the development of inflammation and the activation of other leukocytes, and it also induces apoptosis [23]. The reduction in the level of TNF-alpha of rats treated with HELEPN revealed the anti-inflammatory potential of the extract, which could be due to the presence of phytochemicals in the extract. Saponins have been reported to have a broad range of biological activities, which include anti-inflammation by inhibiting the synthesis and release of inflammatory mediators [24]. Administration of d-galactose also led to increased concentration of Nitric oxide. A high amount of prostaglandins PGE2, cytokines, and nitric oxide (NO) are secreted by the macrophages and other stimulated inflammatory cells [25]. In the male reproductive tract, nitric oxide synthase (NOS) has been found in the testes, epididymis, and vascular endothelial cells. NO in the physiologic level improves sperm motility and function, but excessive NO in sperm causes detrimental effects [26]. Inducible NOS is abundantly expressed in peritubular testicular macrophage, and it is the main source of NO generation in inflammatory conditions [27]. NO reacts with the superoxide dismutase anion produced inside the mitochondria of the germ cells to generate active and strong oxidants [28], which causes high levels of NO unleashes a pathogenic cascade of lipid peroxidation [29] and protein nitration [30]. NO has also been shown to inhibit Leydig cell steroidogenesis directly. HELEPN reduces the concentration of NO, and this further confirms the anti-inflammatory potential of HELEPN. This could be as a result of combining the effects of phytochemicals and minerals present in HELEPN. Alkaloid, which is the most abundant phytochemical in HELEPN, has been reported to have anti-inflammatory activities [31,32], and it could likely attenuate testicular NO and TNF-α release, thus, abrogate galactose-induced oxidative and nitrosative stress, DNA damage, and caspase 3-mediated apoptosis [33].

Notably, inflammatory impairment on the male genital tract causes increased generation of reactive oxygen species (ROS) [34]. The reduction in the production of inflammatory cytokines and nitric oxide by HELEPN, therefore, greatly reduces the secretion of free radicals, thereby preventing oxidative damages to molecules [35].

d-galactose treatment also led to a state of oxidative stress, evident by increased lipid peroxidation and impaired antioxidant buffering capacity, demonstrated by the observed increase in testicular MDA, reduced GSH levels, as well as a decline in GPx, SOD, and catalase activities; this is in tandem with previous studies [18,36]. This study revealed that d-galactose also caused testicular oxidative DNA damage.

The oxidative testicular injury observed was associated with poor sperm quality, possibly due to the susceptibility of the sperm cells to peroxidative damage since they have high concentrations of polyunsaturated fatty acids [37]. The glutathione system is vital in the antioxidant buffering system. GSH forms oxidized glutathione (GSSG) and other disulfides, thus scavenging reactive oxygen species [38]. GPx, another constituent of the glutathione system, is responsible for H_2_O_2_ elimination [39]. Hence, d-galactose-induced decline in GSH/GPx observed in this study might predispose the testis and sperm cells to increased oxidative damage as well as testicular DNA oxidative damage.

In this study, HELEPN significantly suppressed testicular lipid peroxidation, improved GSH levels, and antioxidant activities. Furthermore, the restoration of testicular GSH level and activities of GPx, catalase, and SOD in animals co-treated with HELEPN reveal the antioxidant property of the extract. This agrees with previous studies that reported the antioxidant potential of the extract [40]. The antioxidant potential of HELEPN may be ascribed mostly to alkaloids, saponins, tannins, cardiac glycosides, and anthraquinones content.

This study revealed that the weight of testis, a critical indicator of reproductive impairment, was significantly reduced in animals given d-galactose, and this corroborates previous studies [41,42]. The reduced testicular weight may explain the androgen suppression and poor sperm quality evident by the lowered circulatory testosterone concentration and altered sperm production process in this study. The abnormal sperm morphology due to the administration of d-galactose could also be indicative of reproductive impairment. The relationship between defects in sperm morphology and sperm dysfunction has been established. Abnormal sperm morphology was correlated with infertility, as revealed in the laboratory bioassays [43]. It was also noted that abnormal sperm morphology and multiple deficiencies were observed at the cellular level, such as abnormal basal and stimulated intracellular calcium concentration, abnormal level of creatine kinase, and an increased capability to generate detrimental ROS [44]. This leads to DNA fragmentation and loss of sperm function and to the concept of immature sperm [43]. In addition, weight loss, as observed in d-galactose-treated rats, showed toxicity and disrupted metabolism by the compound [14,45]. Administration of HELEPN led to a significant increase in testicular weight and reinstated the relative testicular weight.

In the present study, d-galactose-induced reduction in serum testosterone concentration is likely due to its ability to induce oxidative stress [46] by the production of free radicals and degeneration of testicular histo-architecture. Administration of HELEPN restored the architectural abnormalities induced in the rat testis. The increased testicular oxidative damage following d-galactose administration, at least in part, accounts for the raised testicular levels of nitric oxide and TNF-α, increased testicular caspase 3 activity, and increased testicular DNA fragmentation observed in the study. This possibly led to peroxidative injury to the testicular macromolecules such as lipids, proteins, DNA, as well as the major enzymes that are used in testicular steroidogenesis and sperm production [47], with a resultant low level of serum testosterone and germ cell maturation arrest.

The reduced level of gonadotropins observed in the present study following d-galactose administration is suggestive of d-galactose-induced suppression of the hypothalamic–pituitary–gonadal axis. Following co-administration of HELEPN, the restoration of serum gonadotropins and testosterone concentration, sperm quality, and low caspase 3 activities were possibly due to HELEPN’s antioxidant effect and restoration of the glutathione buffering capacity. This can also be ascribed to the antioxidant activities of its constituents, especially the phytochemicals and is consistent with the previous study that reported that the extract enhances testosterone biosynthesis [3].

DNA fragmentation is primarily a result of apoptosis or oxidative damage. d-galactose-administered rats treated with HELEPN demonstrated diminished testicular DNA fragmentation associated with attenuation of caspase 3-mediated apoptosis. Apoptosis is a programmed cell death that is tightly regulated. The primary components of apoptosis are groups of proteases called caspases. Caspase-8 and -9, which are the primer caspases, stimulate the activities of the executioner caspases such as caspase-3, -6, and -7, which cause the disintegration of proteins embedded in the cells [48]. Caspase 3 is considered the major executioner caspase [15]. The mitochondrial pathway, which is one of the vital apoptosis-associated pathways, releases cytochrome c from mitochondria, resulting in formation of the apoptosome, consequently leading to activation of caspase 9, which culminates in the activation of caspase 3, resulting in DNA fragmentation [49]. Moreover, the high content of polyunsaturated fatty acid in the cell membrane predisposes the cell to oxidative damage [47], leading to base modifications and DNA fragmentation [50]. Furthermore, ROS activates caspases and endonucleases to induce DNA fragmentation [51]. Hence, the testicular DNA fragmentation observed following d-galactose administration is via oxidative damage as well as caspase-3 dependent apoptosis. This is an indicator that d-galactose may independently trigger oxidative stress and apoptosis. Co-administration of HELEPN attenuated d-galactose-induced oxidative stress and apoptosis, thus highlighting the antioxidant and antiapoptotic potential of HELEPN.

It is likely that the other constituent phytochemicals exert collaborative effects with alkaloids. Saponins stimulate LH and FSH release, which culminates in increased testosterone [3,52]. Polyphenols, such as tanins, have also been known to act as an antioxidant and to enhance reproductive variables [53] and have also been reported to reduce ROS generation, DNA fragmentation, and caspase 3-mediated apoptosis [53], thus enhancing androgen secretion and improving sperm quality. In this study, administration of HELEPN restored the reduced serum testosterone level as well as sperm quality.

## 4. Materials and Methods

### 4.1. Plant Collection

The leaves of *Parquetina nigrescens* were collected from Orita-Naira Area, Ogbomoso, Oyo State, Nigeria. Identification and authentification of the plant material were made by Professor A.T.J. Ogunkunle of the Department of Pure and Applied Biology (Botany unit), Ladoke Akintola University of Technology, Ogbomoso, Nigeria. The name of the plant was confirmed on http://www.theplantlist.org (accessed on 19 August 2019). A voucher specimen (LHO 532) was kept in the Herbarium at the Department of Botany, Ladoke Akintola University of Technology, Ogbomoso, Nigeria.

### 4.2. Preparation of Plant Extract

**Figure 7:**
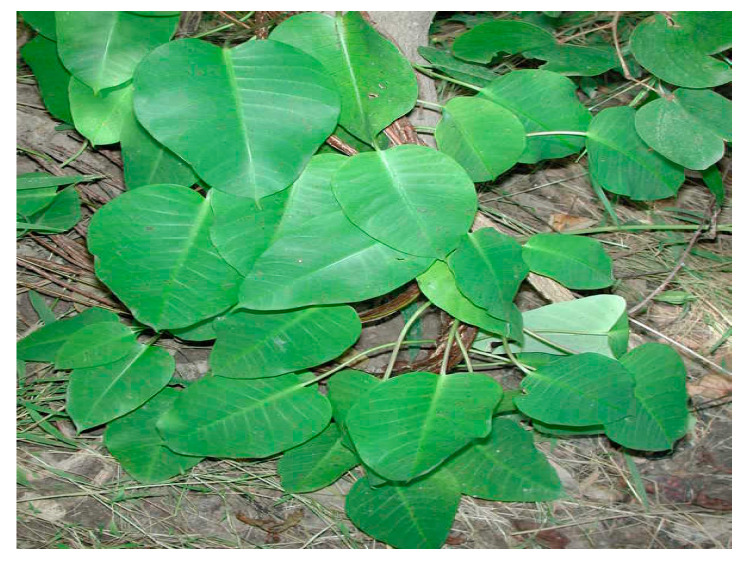
The leaf of *Parquetina nigrescens.*

The leaves of *Parquetina nigrescens* shown in Figure 7 were air-dried for two weeks and blended into powder using an electric blender/mill grate. A 50 g sample was then soaked in 200 mL of hydro-ethanol mixture (ratio 1:2) for 48 h to give a 70% ethanolic extract. The extract was decanted and concentrated using a rotary evaporator. The concentrated extract was then dried using a regulated oven at 45°C [54].

### 4.3. Phytochemical Analysis

Qualitative evaluation of secondary metabolites was carried out using standard methods described by Sofowora [55] and Trease et al. [56], while the quantification was carried out as described in the literature for alkaloids, flavonoids, saponins, phenolics [57,58,59], and tannins [59,60].

### 4.4. Experimental Animals

The experiment was conducted at the animal house of the Department of Biochemistry, Adeleke University, Ede, Nigeria (University Ethical Approval Number: AUERC/FOS/01). Male Wistar albino rats of three months old, weighing between 180–200 g, were used for the study. Wistar albino rats were used for this study because their genetics, biological and behavioral characteristics are closely related to that of humans [61]. The rats were given free access to standard rat chow (Capsfeed, Osogbo, Osun State, Nigeria) and water throughout the experimental period. Experimental procedures followed were in conformity with the instructions for the care and use of Laboratory Animals published by the US National Institutes of Health (NIH Publication No. 85-23, revised 1996).

### 4.5. Experimental Design

The rats were acclimatized for a week, then randomly allotted into six groups, each consisting of five rats. Group 1 (Normal control) was given 1.5 mL of dimethyl sulphoxide (DMSO) as a vehicle [62]. Group II rats served as the d-galactose group and were given 300 mg/kg of d-galactose [63]. Group III rats were given 250 mg/kg of HELEPN [7]. Group IV rats were given 500 mg/kg of HELEPN [7]. Group V received d-galactose, as in group II, as well as HELEPN, as in group III. Group VI rats were given d-galactose, as in group II, as well as HELEPN, as in group IV. d-galactose and HELEPN were administered daily for 6 and 4 weeks, respectively. HELEPN was administered orally, while d-galactose was administered subcutaneously. For groups that received both d-galactose and HELEPN, administration of d-galactose started two weeks prior to that of HELEPN. Dimethyl sulphoxide (DMSO) was used as diluent for the extract.

### 4.6. Blood and Tissue Sample Collection

Body weights of all animals were determined weekly. At the end of the experiment, 24-h fasted rats were anesthetized using ketamine-xylazine (ketamine, 40 mg/kg and xylazine, 4 mg/kg) intraperitoneally [64,65]. Blood samples were taken through cardiac puncture. From each blood sample, 5 mL was centrifuged to obtain the serum, which was kept at 20 °C for the assay of male reproductive hormones. Both testes were removed, dissected, and rinsed in ice-cold saline. One of the harvested testes was weighed and stored in Bouin’s solution for histological examination and the other was homogenized for further analysis.

### 4.7. Antioxidant Indices

Glutathione peroxidase (GPx) was carried out by the method of Rotruck et al. [66], glutathione (GSH) by the method of Moron et al. [67], catalase (CAT) was determined by the method of Aebi [68], while superoxide dismutase (SOD) was determined by the modified method of McCord and Fridovich [69].

### 4.8. Oxidative Stress Biomarkers

Malondialdehyde (MDA) level was determined by the method of Iqbal et al. [70]. Testicular concentrations of Advanced Glycation End products (AGEs) and 8 hydroxydeoxyguanosine (8-OHdG) were assayed using the ELISA kit (Elabscience, Houston, TX, USA) following the manufacture’s guidelines.

### 4.9. Apoptotic Biomarkers

Testicular caspase 3 activity was determined using the ELISA kit (Elabscience, Houston, TX, USA) following the manufacture’s guidelines.

### 4.10. Inflammatory Biomarkers

Testicular level of TNF-α was determined using the ELISA kit (Elabscience, Houston, TX, USA) following the manufacture’s guideline. Testicular level of Nitric oxide was determined as reported by Green et al. [71].

### 4.11. DNA Fragmentation Index Assay (DFI)

DNA Fragmentation Index (DFI) was determined spectrophotometrically using the diphenylamine (DPA) method as described by Perandones et al. [72].

### 4.12. Reproductive Hormones

Serum levels of LH, FSH, and testosterone were measured using standard ELISA kits (Monobind Inc., Lakeforest, CA, USA) following the manufacture’s guidelines.

### 4.13. Sperm Parameters

The left caudal epididymis was separated from the testis and lacerated to collect semen. The epididymis was carefully exposed, and the caudal epididymis was then carefully separated and transferred unto a warm slide (27 °C) and lacerated with a dissecting blade to release some sperm into the slide surface. The sample was analyzed immediately after collection [73].

### 4.14. Histology

The harvested testis were fixed in Bouin and dehydrated sequentially in 50–100% ethanol. Xylene was used to rinse the tissues for removal of dehydrant (ethanol) and then embedded in paraffin for strengthening and easy dissection. The tissues were rinsed in xylene to remove the paraffin before sectioning and washed in decreasing concentration of ethanol (100–50%) before rehydrating the tissue with water. Tissue section of 6 µm thick was made and stained with hematoxylin-eosin (H-E) dye to make it visible for photomicroscopic viewing and then observed under a light microscope at 400× magnification.

### 4.15. Statistical Analysis

The statistical software used for the data analysis was SPSS (version 16). Data were presented as mean ± SEM. The comparison across all the groups was carried out using analysis of variance (ANOVA) followed by Tukey’s post hoc test for pair-wise comparison.

## 5. Conclusions

Overall, this study concludes that HELEPN restored the biochemical aberrations in the rat testis by d-galactose administration. The protective effect of HELEPN was established to be via antioxidative, anti-inflammatory, and antiapoptotic mechanisms, which seem to be mediated, at least in part, by GPx up-regulation and caspase 3 down-regulation.

## Figures and Tables

**Table 1:** Phytochemical Constituents of HELEPN.

Pytochemicals	Values
Alkaloids (mg/g)	72.00 ± 0.45
Saponins (mg/g)	18.00 ± 0.68
Tannins (mg/L)	4.12 ± 0.054
Cardiac glycosides (mg/L)	12.70 ± 0.12
Anthraquinones (mg/100 g)	15.20 ± 0.34

Values are means of three replicates (three analyses of the same sample) ± standard deviation.

**Table 2:** Weight change, Paired Testicular Weight and Relative Testicular Weight.

Group	Initial BW (g)	Final BW (g)	Difference (g)	PTW (g)	PTW/BW × 100
Control	188.00 ± 2.63 ^a^	248.05 ± 1.92 ^a^	60.05 ± 1.93 ^a^	1.04 ± 0.3 ^a^	0.42 ± 0.01 ^a^
d-galactose	192.62 ± 2.21 ^a^	213.80 ± 1.95 ^b^	21.18 ± 2.11 ^b^	0.52 ± 0.33 ^b^	0.24 ± 0.04 ^b^
250 mg/kg HELEPN	193.91 ± 2.19 ^a^	215.09 ± 1.86 ^b^	36.32 ± 2.09 ^b^	1.06 ± 0.42 ^a^	0.49 ± 0.03 ^a^
500 mg/kg HELEPN	189.89 ± 2.11 ^a^	217.21 ± 2.01 ^b^	27.32 ± 1.09 ^b^	1.09 ± 0.36 ^a^	0.50 ± 0.01 ^a^
d-galactose + 250 mg/kg HELEPN	192.34 ± 1.91 ^a^	217.52 ± 1.22 ^b^	25.18 ± 2.13 ^b^	0.99 ± 0.53 ^a^	0.46 ± 0.01 ^a^
d-galactose + 500 mg/kg HELEPN	189.00 ± 1.87 ^a^	213.32 ± 1.67 ^b^	24.32 ± 1.19 ^b^	0.90 ± 0.43 ^a^	0.43 ± 0.01 ^a^

BW, bodyweight of rats; PTW, paired testicular weight. Values are means of five replicates ± SEM. Values carrying different superscripts (a, b) are significantly different at *p* < 0.05.

**Table 3:** Effect of graded doses of HELEPN on sperm morphology.

Parameters	Tailless Head	Headless Tail	Rudiment Tail	Bent Tail	Bentmid Piece	Curved Tail	Curved Midpiece	Looped Tail
Control	4.4 ± 0.8 ^a^	4.6 ± 0.8 ^a^	2.0 ± 1.0 ^a^	9.8 ± 1.0 ^a^	10.0 ± 0.7 ^a^	10.8 ± 0.8 ^a^	10.2 ± 0.4 ^a^	1.8 ± 0.8 ^a^
d-galactose	5.8 ± 0.4 ^b^	5.6 ± 0.5 ^b^	2.5 ± 0.8 ^b^	11.6 ± 1.1 ^b^	11.8 ± 1.5 ^b^	11.6 ± 1.3 ^b^	10.8 ± 0.4 ^b^	2.8 ± 0.4 ^b^
250 mg/kg HELEPN	4.6 ± 1.1 ^a^	2.5 ± 0.8 ^b^	2.0 ± 1.0 ^a^	8.6 ± 1.8 ^a^	9.2 ± 1.4 ^a^	9.0 ± 1.2 ^a^	9.0 ± 1.2 ^a^	1.8 ± 0.8 ^a^
500 mg /kg HELEPN	4.6 ± 0.5 ^a^	4.0 ± 0.7 ^a^	2.0 ± 1.0 ^a^	9.2 ± 1.0 ^a^	9.6 ± 1.1 ^a^	9.6 ± 1.1 ^a^	9.2 ± 0.4 ^a^	1.6 ± 0.5 ^a^
d-galactose + 250 mg/kg HELEPN	4.8 ± 0.8 ^a^	3.8 ± 0.8 ^a^	2.2 ± 0.8 ^a^	9.7 ± 1.3 ^a^	9.6 ± 1.1 ^a^	9.8 ± 0.8 ^a^	7.8 ± 0.8 ^a^	2.0 ± 1.0 ^a^
d-galactose + 500 mg/kg HELEPN	4.6 ± 0.8 ^a^	3.8 ± 0.8 ^a^	2.0 ± 1.0 ^a^	9.0 ± 1.2 ^a^	9.8 ± 1.0 ^a^	9.2 ± 0.4 ^a^	9.4 ± 1.1 ^a^	2.0 ± 1.0 ^a^

Values are means of five replicates ± SEM. Values carrying different superscripts (a, b) are significantly different at *p* < 0.05.

**Table 4:** Effect of graded doses of HELEPN on apoptotic biomarkers.

Group	Testicular DFI (%)	Caspase 3 (ng/mL)
Control	21.8 ± 0.72 ^a^	0.54 ± 0.02 ^a^
d-galactose only	36.2 ± 0.90 ^b^	0.9 ± 0.02 ^b^
250 mg/kg HELEPN Only	18.9 ± 0.20 ^a^	0.46 ± 0.02 ^a^
500 mg/kg HELEPN Only	17.4 ± 0.30 ^a^	0.42 ± 0.07 ^a^
d-galactose + 250 mg/kg HELEPN	28.2 ± 0.83 ^c^	0.52 ± 0.04 ^a^
d-galactose + 500 mg/kg HELEPN	24.4 ± 0.92 ^c^	0.49 ± 0.05 ^a^

Values are means of five replicates ± SEM. Bars carrying different superscripts (a–c) are significantly different at *p* < 0.05; DFI:DNA fragmentation index.

## Data Availability

Not Applicable.

## Sample Availability

Samples of the extract are available from the authors.

## Abbreviations

ANOVA	analysis of variance
SOD	superoxide Dismutase
MDA	Malondialdehyde
GSH	Glutathione
GPx	glutathione peroxidase
HELEPN	hydroethanolic extract of *Parquetina nigrescens*
CAT	Catalase
DNA	deoxyribonucleic acid
LH	leutinizing hormone
FSH	follicle-stimulating hormone
ROS	reactive oxygen specie
AGEs	advanced glycation end products
8-OHdG	8 hydroxydeoxyguanosine
SPSS	Statistical Package for Social Sciences
NO	nitric oxide
DFI	DNA fragmentation index
TNF	tumor necrosis factor
HPLC	high-performance liquid chromatography
ELISA	enzyme-linked immunosorbent assay
